# Development of the Mammary Gland in Mouse: A Whole-Mount Microscopic Analysis

**DOI:** 10.21769/BioProtoc.5089

**Published:** 2024-10-20

**Authors:** Bo Wang, Yuchen Xie, Zejian Yang, Jingyue Zhang, Huiwen Zhang, Peijun Liu

**Affiliations:** 1 Center for Translational Medicine, the First Affiliated Hospital of Xi’an Jiaotong University, Xi’an, Shaanxi, China; 2 Key Laboratory for Tumor Precision Medicine of Shaanxi Province, the First Affiliated Hospital of Xi’an Jiaotong University, Xi’an, Shaanxi, China; 3 Department of Radiation Oncology, the First Affiliated Hospital of Xi’an Jiaotong University, Xi’an, Shaanxi, China; 4 Department of Breast Surgery, the First Affiliated Hospital of Xi’an Jiaotong University, Xi’an, Shaanxi, China

**Keywords:** Mouse, Mammary gland, Whole-mount, Mammary gland development, Morphogenesis, Terminal end buds, Breast cancer, Tumorigenesis

## Abstract

The mammary gland undergoes functional, developmental, and structural changes that are essential for lactation and reproductive processes. An overview of such unique tissue can offer clearer insights into mammary development and tumorigenesis. Compared to traditional methods, mouse mammary gland whole mount is a pivotal technique that provides three-dimensional structural perspectives on gland morphology and developmental stages, offering an inexpensive and accessible approach. This protocol outlines the tissue isolation of the mouse mammary gland and provides detailed instructions for whole-mount staining and analysis. Mammary gland tissues are carefully dissected from euthanized mice and stained with Carmine Alum to highlight the ductal structures, enabling detailed visualization of the branching patterns and morphological changes. Light microscopy is used to capture a panoramic image of the stained mammary gland, enabling the quantitative analysis of terminal end buds (TEBs) and bifurcated TEBs to further investigate mammary gland remodeling. This method can provide invaluable insights, particularly in the study of mammary gland morphogenesis and tumorigenesis, underscoring its significance in both basic research and clinical applications.

Key features

• Monitor mammary gland development within 2 days using whole-mount staining.

## Graphical overview


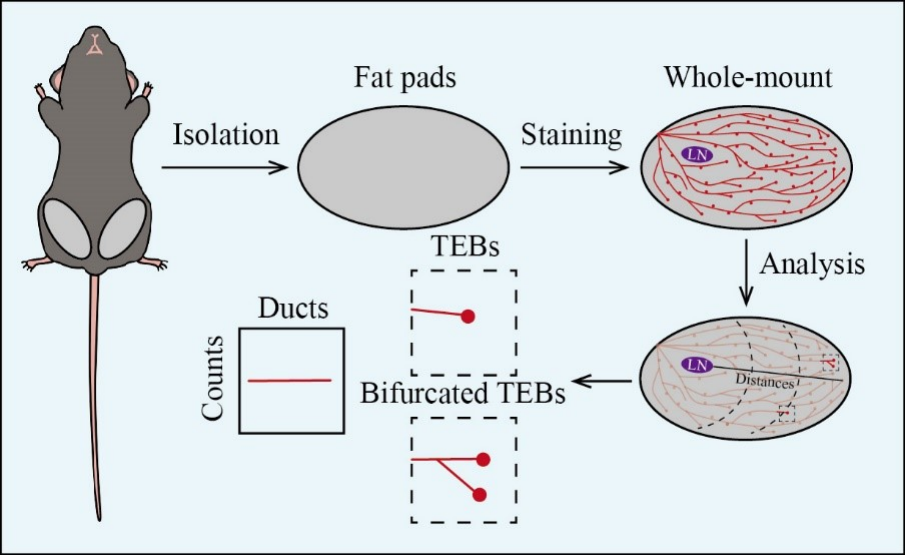


**Mouse mammary gland whole-mount analysis**

## Background

The mammary gland undergoes dynamic developmental stages during the fetus, puberty, and adulthood stages. Developmentally, initial ductal tree formation begins around E10.5, followed by estrogen-induced formation of terminal end buds (TEBs) that drive extensive branching during puberty [1]. The most significant morphogenic event occurs during pregnancy, where lobuloalveolar structures develop at the ends of ducts for milk production [2]. Post-lactation, these structures regress, and mammary glands undergo remodeling. Therefore, the dynamic developmental stages of the mammary gland can be visually observed in three-dimensional structural morphology, which facilitates a better examination of TEBs structure. Presently, classical traditional methods include immunohistochemistry (IHC) and immunofluorescence (IF). For instance, observing the morphology of the mammary gland through hematoxylin and eosin (H&E) staining and specific epithelial cell staining via IHC allows for precise characterization [3]. IF can further label luminal and basal cells to study the functional roles of these cellular subtypes [4]. Mouse mammary gland whole mount, in contrast, is a crucial technique that provides an entire view of duct architecture and developmental processes in mammary gland development and tumorigenesis, without requiring tissue sectioning. It enables the visualization of branching morphogenesis, the formation of TEBs, and the changes in these structures. Therefore, mouse mammary gland whole-mount analysis serves as a powerful tool for advancing our understanding of mammary gland development, pathology, and oncology. This protocol typically includes several key steps: isolation of mouse mammary gland tissue, procedures for whole-mount staining, and subsequent whole-mount analysis. This method provides valuable insights into mammary gland development.

## Materials and reagents

**Biological materials**

1. 8-week-old female mouse

**Reagents**

1. Ethanol (Sinopharm, catalog number: 10009218)
2. Chloroform (Sinopharm, catalog number: 10006818)
3. Glacial acetic acid (Sinopharm, catalog number: 10000208)
4. Carmine (Sigma-Aldrich, catalog number: C1022)
5. Aluminum potassium sulfate dodecahydrate (Solarbio, catalog number: A7520)
6. Hydrochloric acid (HCl) (Sinopharm, catalog number: 10011018)
7. Xylene (Sinopharm, catalog number: 10023418)
8. Permount (Fisher Scientific, catalog number: SP15-500)
9. Neutral balsam (Solarbio, catalog number: G8590)

**Solutions**

1. Carnoy’s fixative (see Recipes)
2. Carmine alum (see Recipes)
3. Wash buffer (see Recipes)

**Recipes**

1. **Carnoy’s fixative (50 mL)**
*Note: Prepare fixative just before use.
  **Table:** 

Reagent	Final concentration	Quantity or Volume
Ethanol	60% (v/v)	30 mL
Chloroform	30% (v/v)	15 mL
Glacial acetic acid	10% (v/v)	5 mL
Total (optional)	n/a	50 mL*
2. **Carmine alum (100 mL)**
*Note: Add approximately 90 mL of water and heat the mixture on a hot plate until it boils for 15 min. After cooling the solution, filter it using filter paper. Adjust the volume to 100 mL and store at 4 °C.
  **Table:** 

Reagent	Final concentration	Quantity or Volume
Carmine	0.2% (w/v)	0.2 g
Aluminum potassium sulfate dodecahydrate	0.5% (w/v)	0.5 g
H_2_O	n/a	see note*
Total (optional)	n/a	100 mL
3. **Wash buffer (100 mL)**
*Note: Add water to make up to 100 mL.
  **Table:** 

Reagent	Final concentration	Quantity or Volume
Ethanol	70% (v/v)	70 mL
HCl (36%–38%)	2% (v/v)	5.64 mL
H_2_O	n/a	see note*
Total (optional)	n/a	100 mL

**Laboratory supplies**

1. Glass slide (Citotest, catalog number: 80312)
2. 50 mL centrifuge tubes (Corning, catalog number: 430829)
3. Coverslip (Citotest, catalog number: 80340)
4. Funnel (Citotest, catalog number: 84112)
5. Filter paper (Citotest, catalog number: 84501)
6. Volumetric cylinder (Citotest, catalog number: 84201)

## Equipment

1. Scissors (RWD, catalog number: S12003-09)
2. Forceps (RWD, catalog number: F12005-10)
3. Autoclave (Panasonic, model: MLS-3781L-PC)
4. Hot plate (IKA, model: C-MAG HP 7)
5. Chemical fume hood (Rista lab, model: RSD-BLGTFG)
6. Dissecting microscope (OLYMPUS, model: SZX7)

## Software and datasets

1. Prism v9.3 (GraphPad, 11/15/2021)
2. ImageJ 1.53e (NIH)

## Procedure

- **Isolation of mouse mammary gland tissue**
  1. Euthanize an 8-week-old female mouse using CO_2_ inhalation.
  2. Secure the limbs of the mice to a foam board with pushpins.
  3. Using sterile scissors, make a skin incision on the abdomen and extend it toward the midline and hind limbs without breaching the peritoneum (Figure 1A).
    **Figure 1.**
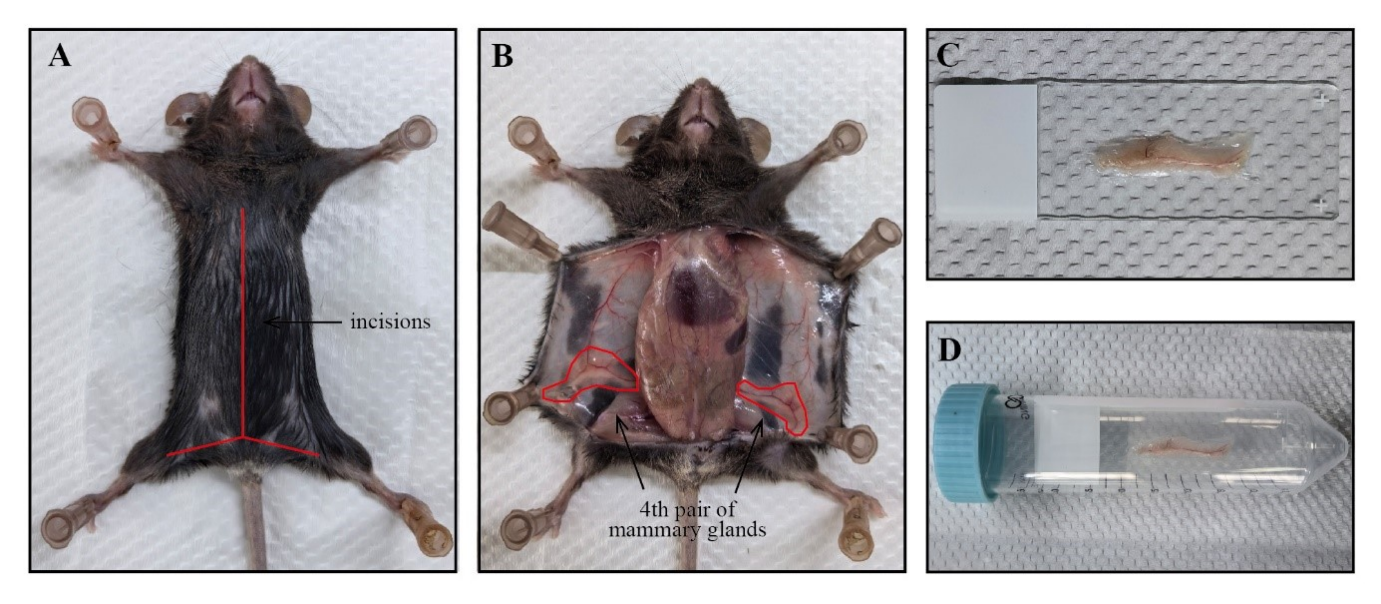
Isolation of mouse mammary gland tissue for whole-mount staining. A. The euthanized mouse is secured on a foam board, and its skin is incised along the midline (as indicated by the red line). B. The incised skin is pinned back to fully expose the fourth pair of mammary glands (as marked within the red circle). C. The entire mammary gland tissue is flattened on a glass slide. D. The glass slide is inserted into a centrifuge tube for staining.
  4. Securely pin the opened skin flat onto the foam board to adequately expose the fourth pair of mammary glands (Figure 1B).
  5. Gently grasp the distal end of the entire fat pads using sterile forceps and slowly remove the mammary glands from the skin using sterile scissors. For detailed steps, refer to the video "Skin incision, removal of the mammary gland and spreading of the tissue onto glass slides" in [5].
  6. Transfer the entire tissue onto a glass slide and gently spread the tissue using forceps (Figure 1C).
  7. Place the glass slide at room temperature (RT) and allow the tissue edges to dry. After confirming that the tissue is securely adhered and not easily detached, transfer it into a 50 mL centrifuge tube for subsequent whole-mount staining (Figure 1D).
- **Whole-mount staining**
  1. Slowly add newly prepared Carnoy’s fixative to a 50 mL centrifuge tube, ensuring complete immersion of the entire mammary gland tissue in the chemical fume hood for 2–4 h or overnight at RT (Figure 2A).
    **Figure 2.**
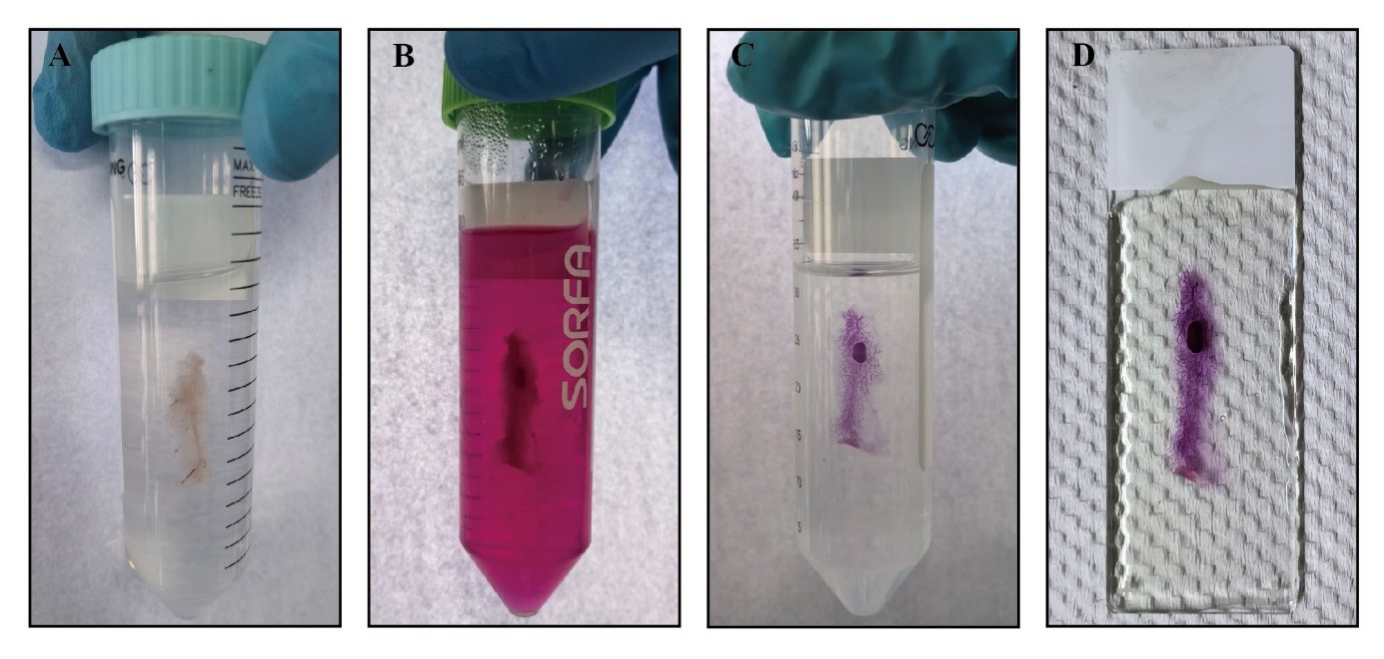
Process of whole-mount staining of mouse mammary gland tissue. A. The entire mammary gland tissue is fixed in Carnoy’s fixative. B. The entire mammary gland tissue is stained in Carmine Alum. C. The stained mammary gland tissue is cleared in xylene. D. The cleared mammary gland tissue is covered in neutral balsam.
  2. Transfer the glass slides to a 50 mL centrifuge tube for washing with 70% ethanol in the chemical fume hood for 15 min at RT. Mammary gland tissue processed in this step can be stored for several months.
  3. Pass the glass slides sequentially through 50% and 30% ethanol and ddH_2_O in the chemical fume hood for 5 min each at RT.
  4. Transfer the glass slides to Carmine Alum in the chemical fume hood at 4 °C overnight. Adjust staining duration based on the size and thickness of mammary gland tissue. Completed staining reveals a carmine-colored mammary epithelial tree and lymph node (Figure 2B).
  5. Wash the glass slides with wash buffer in the chemical fume hood at RT until the solution becomes clear.
  6. Pass the glass slides sequentially through 70%, 95%, and 100% ethanol in the chemical fume hood for 15 min each at RT.
  7. Transfer glass slides to xylene for tissue clearing (Figure 2C) and mount them with Permount in the chemical fume hood at RT.
  8. Apply Permount or neutral balsam to cover the entire mammary gland tissue in the chemical fume hood (Figure 2D) and store at RT.
- **Whole-mount analysis**
  1. Capture images of the stained entire mammary gland tissue using a dissecting microscope (Figure 3A).
    **Figure 3.**
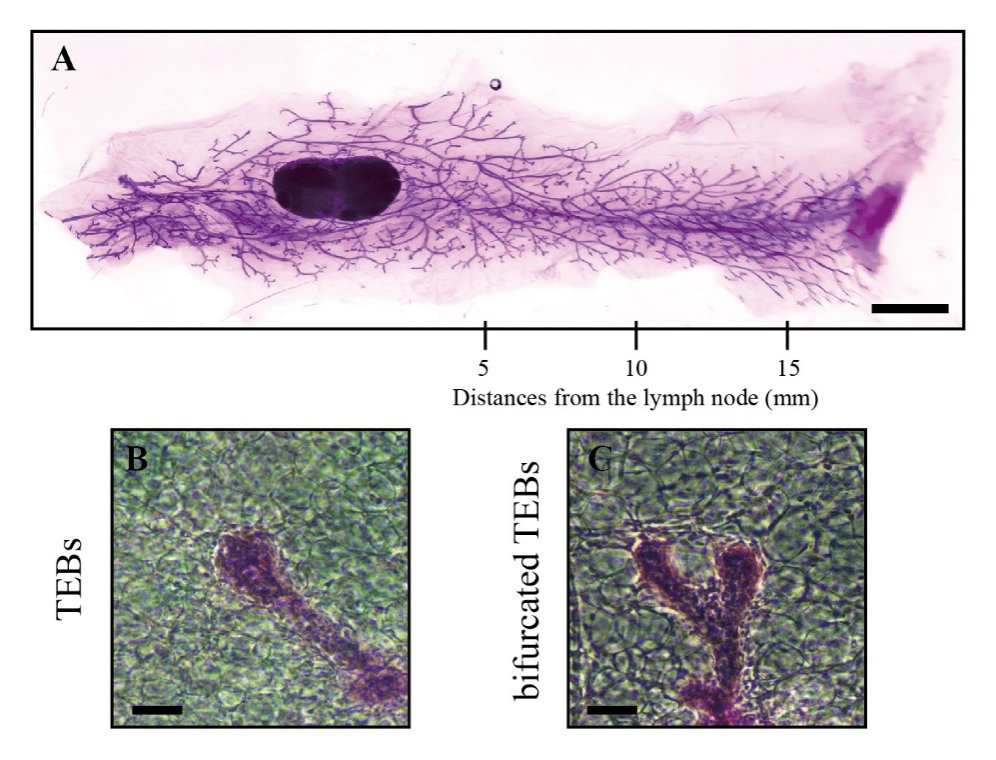
Information from whole-mount analysis of mouse mammary gland tissue. A. The stained entire mammary gland tissue was captured by dissecting microscopy and contains information at different distances from the lymph node (scale bars, 2.5 mm). B–C. Images of TEBs (B) and bifurcated TEBs (C) were enlarged from the panoramic image (scale bars, 100 μm).
  2. Count the number of TEBs (Figure 3B) and bifurcated TEBs (Figure 3C) directly on the images using a counter.
  3. Count the number of ducts and acini at distances of 5, 10, and 15 mm from the lymph node along the direction of the mammary gland (Figure 3A).

## Data analysis

**Figure 4.**
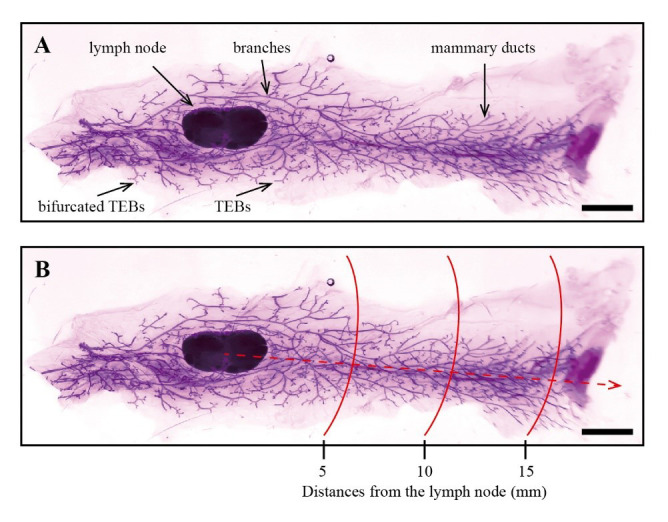
Examples of whole-mount analysis of mouse mammary gland tissue. A. Examples of whole-mount staining of mouse mammary gland tissue. B. Measurements of 5, 10, and 15 mm lengths (as marked within the red lines) were taken starting from the lymph node by using ImageJ (scale bars, 2.5 mm).

The development of the mammary gland can be assessed directly by evaluating the mammary epithelial tree. Within this panoramic image, structures such as lymph nodes, TEBs, mammary ducts, and branches of the fourth pair of mammary glands can be distinguished (Figure 4A).

Prism was utilized to plot and statistically analyze the counts of TEBs and bifurcated TEBs within the entire mammary gland tissue, typically represented as "TEBs/gland (n)" and "bifurcated TEBs (n)."

Using ImageJ, the *Straight* tool was employed to draw scalebars length, and the scale was set using *Analyze* > *Set Scale* with the known distance input for the actual measurement of scale bars, ensuring *Global* was selected. Measurements of 5, 10, and 15 mm lengths were taken starting from the lymph node (Figure 4B). Prism was used to plot and statistically analyze the counts of ducts and acini within the 5, 10, and 15 mm distances from the lymph node, as well as across all distances, typically represented as "average complexity."

## Validation of protocol

This protocol or parts of it has been used and validated in the following research article(s):

- Wang et al. [7]. CRB3 navigates Rab11 trafficking vesicles to promote γTuRC assembly during ciliogenesis. eLife (Figure 1, panel C).

## General notes and troubleshooting

**General notes**

1. Mouse mammary gland whole mount is suitable for analyzing different stages of mammary gland development in mice, including pre-pubertal (5 weeks old), pubertal (8 weeks old), pregnant, and aging (25–90 weeks of age) stages.
2. A 50 mL centrifuge tube can be used to stain slides of two mammary gland tissue samples placed back-to-back.
3. To a better demonstration of mouse mammary glands whole mount from various groups, we attempted to parallelly lay out two whole mammary gland tissues on a single slide for subsequent staining and analysis.
4. If Permount is not readily available, mammary gland tissue clearing can be achieved using xylene, followed by mounting with neutral balsam.
5. Due to the thickness of the entire mammary gland tissues, the coverslip may not always sit flat during mounting. Applying pressure can flatten the coverslip and ensure a smooth seal.

**Troubleshooting**

Problem 1: The stained mammary gland tissues are inadequately stained or appear faintly stained.

Possible cause: Insufficient staining time.

Solution: Increase the staining duration, potentially allowing the mammary gland tissue to stain in Carmine Alum for several days.
